# Current Status of Mining, Modification, and Application of Cellulases in Bioactive Substance Extraction

**DOI:** 10.3390/cimb43020050

**Published:** 2021-07-13

**Authors:** Yawei Hu, Guangbo Kang, Lina Wang, Mengxue Gao, Ping Wang, Dong Yang, He Huang

**Affiliations:** 1 Department of Biochemical Engineering, School of Chemical Engineering & Technology, Tianjin University, Tianjin 300350, China; huyawei@tju.edu.cn (Y.H.); guangbo_kang93@tju.edu.cn (G.K.); m01781299@tju.edu.cn (L.W.); 2018207213@tju.edu.cn (M.G.); 2 Frontiers Science Center for Synthetic Biology and Key Laboratory of Systems Bioengineering (Ministry of Education), School of Chemical Engineering & Technology, Tianjin University, Tianjin 300072, China; dongyang@tju.edu.cn; 3 Tianjin Modern Innovative TCM Technology Co. Ltd., Tianjin 300392, China; pingp-w@163.com; 4 School of Environmental Science & Engineering, Tianjin University, Tianjin 300072, China; 5 Zhejiang Shaoxing research Institute, School of Chemical Engineering & Technology, Tianjin University, Shaoxing 312300, China

**Keywords:** cellulase, mining, modification, extraction, medicinal plants, bioactive substances

## Abstract

Cellulases have been used to extract bioactive ingredients from medical plants; however, the poor enzymatic properties of current cellulases significantly limit their application. Two strategies are expected to address this concern: (1) new cellulase gene mining strategies have been promoted, optimized, and integrated, thanks to the improvement of gene sequencing, genomic data, and algorithm optimization, and (2) known cellulases are being modified, thanks to the development of protein engineering, crystal structure data, and computing power. Here, we focus on mining strategies and provide a systemic overview of two approaches based on sequencing and function. Strategies based on protein structure modification, such as introducing disulfide bonds, proline, salt bridges, *N*-glycosylation modification, and truncation of loop structures, have already been summarized. This review discusses four aspects of cellulase-assisted extraction. Initially, cellulase alone was used to extract bioactive substances, and later, mixed enzyme systems were developed. Physical methods such as ultrasound, microwave, and high hydrostatic pressure have assisted in improving extraction efficiency. Cellulase changes the structure of biomolecules during the extraction process to convert them into effective ingredients with better activity and bioavailability. The combination of cellulase with other enzymes and physical technologies is a promising strategy for future extraction applications.

## 1. Introduction

**Figure 1:**
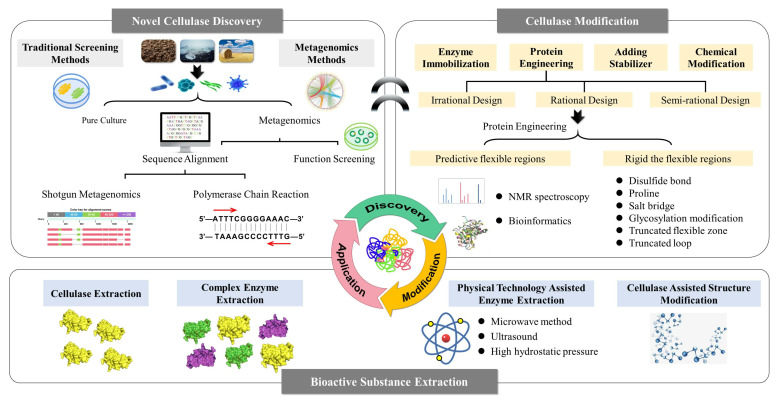
Strategies for obtaining high-performance cellulase and its application in natural medicine extraction.

Cellulose is a macromolecular polysaccharide linked by glucose via a β-1,4-glycosidic bond, is insoluble in water and organic solvents, and forms the plant cell wall together with hemicellulose, pectin, and lignin [1]. Cellulases are a group of enzymes that can hydrolyze the glycosidic bonds of cellulose to produce glucose, and the members of this group include exoglucanase, endoglucanase, and β-glucosidase. The synergistic action of these enzymes can break down cellulose into glucose. Endoglucanase cuts randomly at the cellulose polysaccharide chain’s internal sites to generate oligosaccharides of various lengths and new chain ends, exoglucanase acts on the reducing or nonreducing ends of the cellulose polysaccharide chain to release glucose or cellobiose, and β-glucosidase hydrolyzes cellobiose to form glucose [2]. Cellulases are already widely used in various biological industries, including food, wine, animal feed, laundry, pulp, and agriculture. In the food industry, cellulase can be used to extract fruit and vegetable juice, produce nectar and fruit puree, etc. The use of cellulase in the wine industry can decompose starch and cellulose into sugar and increase the wine yield. Cellulase is also a feed additive, which can reduce the nutrient loss of feed and promote digestion and absorption. Cellulase can also be added to washing powder to enhance the performance of detergents, remove small and fuzzy fibrils on the surface of the fabric, and improve appearance and color brightness. Cellulase can improve the drainage, beating, and running properties of paper mills. The mixture and separated components of cellulase, hemicellulose, and pectinase have potential applications in agriculture, and can be used to control plant diseases and promote plant growth and development [3]. Due to the continuous consumption of fossil fuels, the shortage of energy has become a global problem, accompanied by serious environmental pollution and global warming. There is therefore an urgent need to develop alternative energy sources to reduce dependence on fossil fuels and ease environmental stress. The biochemical conversion of biomass mainly includes three steps: first remove lignin and hemicellulose, then decompose cellulose into glucose, and finally ferment with glucose to produce ethanol. Cellulases and other enzymes can be used to transform natural renewable biomass (such as agricultural and forestry waste) into biofuel [4]. Cellulases can also be used to extract bioactive ingredients for natural medicines. Traditional methods for extracting and separating bioactive ingredients, such as decoction, dipping, percolation, and reflux, all have their shortcomings. Low extraction rate, high impurity content, energy consumption, and long production cycles directly restrict the development of the pharmaceutical industry. Alongside the rapid development of modern industrial engineering technology, novel technologies have been continuously applied to natural medicine production to improve extraction efficiency [5]. Enzymes have been used since the mid-1990s to extract and separate traditional natural medicines. Although enzyme use in the traditional natural medicine pharmaceutical industry started late, it has since been shown to have unique advantages and broad application prospects [6]. Cellulases are widely used in the extraction of natural medicine because they can destroy plant cell walls and facilitate bioactive ingredient extraction. The catalytic activity of any cellulase depends on its spatial structure and is often affected by any physical and chemical factors that can lead to denaturation and inactivation. Although research on cellulase-assisted extraction technology has made rapid progress, its focus is mainly on the exploration of process conditions using existing enzymes. The lack of cellulases with new functional properties has become the bottleneck in cellulase-assisted extraction of natural active substances. Current research focuses on finding new and efficient cellulases suitable for economical industrial production. This review discusses the strategies to obtain novel cellulases based on sequence alignment and gene function screening, methods to improve cellulases performance through protein engineering, and the application of cellulase in bioactive substance extraction (Figure 1).

## 2. Methods to Optimize Cellulase Performance

### 2.1. Cellulase Gene Mining

The traditional gene mining method is based on culturing environmental samples in a medium and then selecting a single colony to make a pure culture. The enzyme activity of the single colony is verified, and the enzyme gene is cloned into a commonly used host for expression [7]. Since most microorganisms (over 99%) in environmental samples cannot be obtained using traditional isolation and culture methods, a large amount of genetic information in the environment is ignored by this approach [8]. With the development of sequencing technology, metagenomics (the genomic analysis of microorganisms by directly extracting and cloning DNA from an assemblage of microorganisms, also referred to as environmental and community genomics) is applied in gene mining to overcome these shortcomings [9]. At present, there are two main metagenomics methods for finding new enzyme genes in environmental samples [10] (Table 1).

#### 2.1.1. Sequence Alignment-Based Methods

With shotgun metagenomic sequencing, all genes from the total DNA obtained in environmental samples are sequenced then annotated according to the existing database, such as Nr, KEGG, and EggNOG [23]. Any novel functional genes with a certain degree of similarity to existing annotated cellulase genes are expressed by transforming them into E. coli and then verifying whether they have cellulase activity. This method significantly improves the efficiency of mining new enzyme genes [24]. 

There are also PCR-based gene mining methods. First, degenerate primers based on the conserved regions of amino acid sequences of known cellulase are designed. Then, the total DNA from the sample is used as a template to perform PCR to obtain target genes with similarity to the known sequence. These genes are then transformed into *E. coli* or other hosts for expression and to verify whether they have cellulase activity [25]. 

Chemical synthesis can be also used to synthesize unverified genes in existing databases, and then functional verification can be performed [26]. Other methods include the transposon-aided capture method to capture novel plasmid in the total DNA of a sample and designing primers based on the integron conserved sequences to obtain novel genes from integron-gene cassettes [27].

#### 2.1.2. Gene Function Screening-Based Methods

Many genes that have been sequenced cannot be accurately annotated due to the limited specific information in existing databases. Novel gene mining methods based on gene function do not rely on existing databases and can therefore discover new genes and/or gene functions. This method needs a metagenomic library to be constructed. The general process involves digestion of the environment’s total DNA to obtain certain lengths of DNA fragments, connecting them with a suitable vector, and then transferring them into a selected host for gene expression and cellulase activity verification [28]. 

Although some new cellulase genes, such as Cel5A, CelA2, and CelA3, have been successfully screened this way, it is difficult to meet the rapidly growing industrial demand due to the high workload and low success rate of library construction. A method based on metagenomic sequencing and subsequent expression verification can significantly improve new gene mining efficiency, and the continued development of this technology provides new ideas for mining novel genes in extreme environments [29].

### 2.2. Rational Design to Improve Cellulase Thermostability (and Specific Activity)

Enzyme reaction conditions are relatively mild and high temperatures will denature the enzyme and render it inactive. Bioactive substance extraction is mostly carried out in higher temperature conditions, which limits enzyme usage to a certain extent. It is therefore necessary to explore strategies to improve the thermal stability of the enzyme [30]. Common strategies include enzyme immobilization [31], addition of stabilizers [32], chemical modification [33,34], and protein engineering [35]. Protein engineering involves modifying proteins at the molecular level and includes rational design, semirational design, and irrational evolution [36]. Of these, rational design, with its high efficiency and strong versatility, has attracted more attention from researchers. To improve enzyme thermostability through rational design it is necessary to analyze the enzyme structure and determine the regions related to the thermostability. If the selected area is not appropriate, it may damage the structure after modification, thereby affecting enzyme activity or even reducing the thermostability. By comparing the structures, thermophilic enzymes were found to be more rigid than mesophilic enzymes, which may be due to the presence of more hydrogen bonds, disulfide bonds, salt bridges, or hydrophobic interactions [37,38]. Similarly, in comparisons of mesophilic and psychrophilic enzymes, it was found that psychrophilic enzymes are more flexible to allow the easy transformation of substrates at low energies [39]. As the highly flexible region of the protein is the first to unfold at high temperatures, flexibility can be used as an indicator to determine potential areas for modifying and improving enzyme thermal stability. Most current studies use site-directed mutations in flexible regions to increase protein rigidity and thermal stability [40].

#### 2.2.1. Prediction of Flexible Regions in Cellulase

The flexible regions of proteins can be predicted either through experimental (such as nuclear magnetic resonance spectroscopy) or bioinformatics methods [41]. With the advances in computational experimental data acquisition and analysis, algorithm optimization, and computing power, researchers have developed a range of bioinformatics software to analyze protein structure and predict flexible regions. Some programs used for predicting protein flexibility are listed in Table 2, and the most common approaches use molecular dynamics simulation and B-FITTER.

#### 2.2.2. Methods for Stiffening Flexible Regions

After identifying flexible regions, several strategies can be used to rigidize the region and improve thermostability. These include the introduction of disulfide bonds, proline, or salt bridges; *N*-glycosylation modification; and the truncation of flexible regions of loop structures (Table 3).

**Figure 2:**
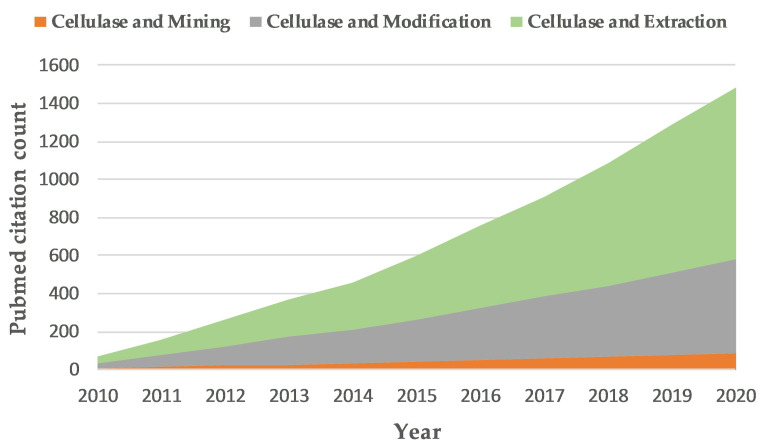
PubMed citation count of cellulase research by year.

The sulfhydryl groups (-SH) in two cysteines can be oxidized to form a disulfide bond which could increase thermostability by reducing the conformational entropy in the unfolded state of the protein and increasing the free energy. Introduction of disulfide bonds into flexible regions can stiffen it and improve protein thermostability [49]. Similarly, proline introduction is another strategy to improve thermostability based on “entropy stabilization” [50]. Of the 20 naturally occurring amino acids, proline has the most robust rigidity, and therefore, during protein unfolding, it will simultaneously reduce the conformational entropy of the main chain and increase the thermal stability [51]. Reducing conformational entropy can also be achieved by truncating the flexible region, which may also lead to an increase in thermal stability [52,53]. Flexible regions are usually located at the *N*- and *C*-terminal ends of a protein, or in the random coil structure. Several studies have shown that salt bridges play an essential role in protein thermal stability and that the numbers of bridges are positively related to thermostability [54]. During protein synthesis, post-translational modifications are crucial, and different modification processes will have different effects on proteins. More than half the proteins in nature are glycosylated [55]. Protein glycosylation is where one or more sugar chains are linked to the protein through covalent interaction. Glycosylation modifications are generally divided into two categories based on the sugar chain connection site: *N*- [56] and *C*-glycosylation [57]. *N*-glycosylation modification accounts for over 75% of modifications. Glycosylated proteins are less likely to aggregate and prevent hinges or links from being affected, and their thermal stability can be improved. Compared with conventional secondary structures, such as α-helix and β-sheet, loops contain fewer hydrogen bonds, resulting in a more flexible region. To explore the relationship between loop structure and protein thermostability, researchers compared the structures of thermophilic enzymes and mesophilic homologs. Studies indicated that the loop structure of thermophilic enzymes may be obtained by truncating part of the loop structure in mesophilic homologs [58]. It was shown that loop length was negatively correlated with thermal stability [59]. In 1999, Thompson proposed that truncating the loop structure of a protein would reduce its conformational entropy and increase stability [60]. At present, some studies introduce site-directed mutations into the flexible loop structure to improve the thermal stability of proteins (Figure 2).

## 3. Cellulase Use in Extraction of Natural Active Substances

Many plants contain various bioactive components with medicinal value. The key to scientific research and effective use of these bioactive substances is to develop effective extraction methods. The limitations of traditional extraction and separation methods, such as low extraction efficiency, low impurity removal rate, high energy consumption, and long production cycle, directly restrict pharmaceutical industry development. Solvent extraction is the most widely used traditional method. However, several components are challenging to effectively extract this way since some bioactive substances are acidic or alkaline, have poor solubility, or interact with other bioactive ingredients [61]. Previous studies have shown that with traditional solvent extraction methods, appropriate addition of acids, bases, surfactants, enzymes, or other extraction aids can improve extraction efficiency and increase extracted component solubility. Some impurities can also be removed or reduced, and preparation stability can be increased [62].

Plant cell walls are dense structures composed of cellulose, hemicellulose, pectin, and lignin. Most bioactive ingredients in plants exist in cells, with a small amount found in the intercellular space. Enzymatic extraction selects enzymes with high specificity based on cell wall composition to directly target the cell wall and destroy its structure. This method can fully expose, dissolve, or suspend the bioactive ingredients in the solvent, thereby extracting bioactive ingredients from plant cells. Enzyme-assisted extraction improves the extraction efficiency, shortens the extraction time, can reduce the destruction of pharmaceutical ingredients, and is suitable for extracting heat-sensitive and unstable chemical components. Maintaining enzyme activity requires strict reaction conditions such as temperature and pH to avoid enzyme inactivation during the extraction process. During enzyme-assisted extraction, the type and amount of enzyme, extraction temperature, time, and pH will all have different degrees of influence on extraction efficiency. The ratio of different enzymes is another important factor when using complex enzymes for extraction. It is therefore necessary to research and select the appropriate technology and conditions.

### 3.1. Structural Modification of Bioactive Ingredients Using Cellulase

The poor water solubility, permeability, or stability of some bioactive ingredients lead to their low bioavailability and poor therapeutic effect, so their application in food and medicine is limited. Enzymes can be used to transform the structure of bioactive ingredients while improving physical and chemical properties and bioavailability. For example, hydrolase can hydrolyze the glycosidic bonds of flavonoid glycosides, and glycosyltransferase and glycosidase can add sugar groups to flavonoids. A single enzyme, or the entire microbe, can be used for biocatalytic oxygenation to hydroxylate flavonoids. The utilization of enzyme modification and promotion of cell wall degradation provides a new method for extracting natural compounds from plants. 

Chang et al. optimized conversion of ginseng saponin glycosides to 20(S)-ginsenoside Rg 3 using the response surface methodology (RSM) and found that cellulase-12T was the most efficient at producing 20(S)-ginsenoside Rg 3. The results indicate that white ginseng extract (WGE) (1.67%) treated with Cellulase-12T (3.67%) for 72 h had 4 times quantity of 20(S)-ginsenoside Rg 3 compared to commercial white ginseng extract [63]. Winotapun et al. developed a method to directly produce Genipin (an iridoid aglycone) from gardenia fruit relying on cellulases to destroy plant cells and cleave off sugar molecules, thereby enhancing the release of intracellular iridoids and converting geniposide into Genipin. Experiments showed that after the crude gardenia fruit was incubated with cellulase (10 mg/mL) at pH 4 for 24 h at 50 °C, in-situ extraction of Genipin with a yield of 58.83 mg/g could be obtained. Compared to the yield obtained by methods that do not require enzymes or in situ extraction, this is an increase of 12.38 and 1.72 times [64]. Chen et al. reported a new method to improve flavonoid extraction from ginkgo leaves using Penicillium decumbens cellulase, a commercial cell wall degrading enzyme with high transglycosylation activity which results in better extractions than Trichoderma reesei cellulase and Aspergillus niger pectinase, and can transglycosylate flavonoid aglycones into more polar glucosides. Transglycosylation has similar optimal conditions to the enzyme-assisted extraction for the three main flavonoids in Pseudomonas ginkgo. The final extraction yield was 28.3 mg/g dry weight (dw), 31% higher than the pre-optimized conditions and 102% higher than enzyme-free conditions [65]. Palaniyandi et al. combined high hydrostatic pressure (HHP) and enzymes to develop a simultaneous extraction and transformation process, to increase the yield of ginsenoside Rd. They found that under the following conditions; pH 4.8, 45 °C, enzyme combination of cellulase (2 U/mL) and cellobiase (4 U/mL), and at HHP (100 Mpa) for 24 h, the ginsenoside Rd content was 3.47 ± 0.35 mg/g fresh ginseng. This yield is 2.1 times that of the same enzyme treatment under atmospheric pressure conditions (AP, 0.1 Mpa). This simultaneous extraction and transformation process can be used to prepare Rd-rich ginseng beverages without using dangerous organic solvents [66] (Table 4).

### 3.2. Extracting Bioactive Ingredients Using Cellulase Alone

Cellulase is widely used to degrade the cellulose in the cell wall, thereby destroying its structure and fully extracting the effective ingredients in the cell. Pan et al. optimized the enzyme-assisted extraction technology of *Dendrobium chrysostom* polysaccharides (DCP) and studied the physical, chemical, and functional properties of DCP-E obtained by enzyme-assisted extraction and DCP-H obtained by hot water extraction. The best conditions for DCP-E extraction are pH 5.5, 40 °C, cellulase at 10 g/L, extraction time of 3.0 h, and a solid–liquid ratio of 1:25. Under these conditions, the DCP-E yield is 8.41 g/100 g dw, 1.25 times that of DCP-H. Compared with DCP-H, DCP-E had a higher purity and cell proliferation rate and a lower molecular weight and relative viscosity [67]. Liu et al. proposed a new method for enzyme-assisted extraction of chlorogenic acid from *Eucommia ulmoides* in an ionic liquid aqueous medium. Compared to other conventional extraction techniques, this method provided advantages in terms of yield and efficiency. Scanning electron microscopy of plant samples showed that cell wall treatment with cellulase in an ionic liquid solution achieved a higher extraction efficiency by reducing mass transfer barriers [68]. Zhang et al. used Congo red staining to identify three *Angelica* endophytes with higher cellulase activity, of which, No.Lut1201 increased Z-ligustilide extraction 2-fold compared to commercially available cellulase (Ningxia Sunson) using a cellulase-assisted extraction method. The cellulase extracted from endophytes enhances cell wall polysaccharide degradation as well as Z-ligustilide extraction from Radix *Angelica sinensis* [69]. Cao et al. used a cellulase-assisted method to extract crude *Astragalus* polysaccharide (APS) from *Astragalus* and analyzed the monosaccharide components of deproteinized APS. Compared with the water extraction method, the cellulase-assisted extraction increased crude APS yields to 154% and polysaccharide content to 121%. The monosaccharide composition of the APS was changed and the galacturonic acid content increased significantly [70]. Park et al. used *Bacillus amylolus* DL-3 cellulase to extract reducing sugars from the fruit of *Hovenia dulcis*, which increased sugar release and reduced extraction temperature and time. The yield of reducing sugar was 1.43 times higher with cellulase than without [71]. 

When using cellulose-rich plant roots, stems, bark, etc. as raw materials, proper use of cellulase treatment can change the cell wall to varying degrees, such as softening, swelling, and collapse. It can improve the permeability of the cell wall, which is conducive to the dissolution of bioactive ingredients, thereby increasing the yield. Mild cellulase hydrolysis conditions reduce the difficulty of subsequent solvent extraction, help maintain the original properties of the bioactive ingredients, and improve the purity (Table 5).

### 3.3. Extraction of Bioactive Ingredients Using Complex Enzymes

Extraction methods using complex enzymes (made by mixing different types of enzymes such as cellulase, pectinase, and protease, in appropriate ratios) have been recently used to extract bioactive ingredients from medicinal plants. Research into optimal conditions such as enzyme ratio and quantity, temperature, and pH is generally performed by orthogonal experiments or RSM based on the optimal conditions of a single enzyme. The advantage of complex enzyme extraction is that it can simultaneously degrade different cell wall components and improve extraction efficiency. Its use has been reported in the extraction of polyphenols, polysaccharides, saponins, and other components. Su et al., evaluated the efficiency of different enzymes (protease and cellulase) to extract rosmarinic acid from *Salvia miltiorrhiza* leaves using an aqua-enzymatic method. Their results showed that a mixture of cellulase A and Protamex (1:1, *w*/*w*) was effective in extracting rosmarinic acid (final yield of 28.23 ± 0.41 mg/g) under the following conditions: enzyme loading rate of 4.49%, water/sample ratio of 25.76 mL/g, 54.3 ℃, and extraction time of 2 h [77]. Chen et al. used RSM and orthogonal experiments to optimize the conditions for extracting APS with combined enzyme extraction. They found that the best extraction conditions were a mix of cellulase (1.5%), pectinase (1%), and papain (0.5%) and an extraction time of 94.5 min at 49.9 ℃ and pH 5.1. Under these conditions, the APS extraction rate was 3.8%, an increase of 52% compared to reflux extraction. The combined enzymatic hydrolysis also reduced the molecular weight of APS, increasing its antioxidant activity [78]. Olivares-Molina et al., used two extraction methods, enzymatic (cellulase and α-amylase) and conventional (impregnation), to maximize extraction yields from three brown seaweeds, *Lessonia nigrescens* (in two stages of development), *Macrocystis pyrifera*, and *Durvillaea antarctica*. The extracts were evaluated as a natural inhibitor of angiotensin I converting enzyme, and the one produced by macerating extraction was a less effective inhibitor than that produced by enzymatic extraction [79]. Zhao et al. optimized enzyme-assisted extraction conditions of polysaccharides from *Lentinus edodes* (LEPs) using cellulase, papain, and pectinase at 15, 20, and 15 g/kg, respectively. They used Box–Behnken design to evaluate and optimize the impact of extraction conditions and found that the highest polysaccharide yield (15.65%) occurred under the following conditions: 54 °C, pH 5.0, for 93 min with a liquid/material ratio of 29:1 mL/g [80]. Lei et al., studied the effects of cellulase, pectinase, and xylanase on the yield of polysaccharides through single-factor experiments and determined the optimal conditions for extracting polysaccharides from white hyacinth beans. They showed that pH (*p* = 0.0599), cellulose (*p* = 0.0756), and water-to-substance ratio (*p* = 0.0951) are important factors for extracting polysaccharides. Other factors affecting polysaccharide yield are cellulose, pectinase, xylanase, water-to-material ratio, extraction temperature, time, and pH. They showed that the optimal conditions for polysaccharide extraction are pH 7.8, cellulose content of 2.7%, and a water-to-material ratio of 62. Under these conditions, the polysaccharide yield was 3.23% [81]. Song et al. used enzymes to assist in extracting functional polysaccharides from Korean ginseng (*Panax ginseng* Meyer) and studied its physical, chemical, and biological properties. The polysaccharide extracted with cellulase and α-amylase contained a higher proportion of pectin polysaccharides with enhanced immunostimulatory properties [82]. Yasutaka et al. studied the method of extracting essential oils from menthol with three polysaccharide degrading enzymes (cellulase A “Amano” 3, cellulase T “Amano” 4, and hemicellulase “Amano” 90). Compared to enzyme-free extraction, 2 wt% cellulase T and 2 wt% hemicellulase 90 for 3 h increased the amount of essential oil extracted from 2.2 to 3.0 mL [83]. Nguyen et al. reported a novel targeted enzyme-assisted method for extracting fucoidan from brown algae involving the combined use of cellulase and alginate lyase from *Sphingomonas* sp. at pH 6.0 and 40 °C, with the removal of non-fucoidan polysaccharides by Ca^2+^ precipitation and ethanol precipitation of crude fucoidan [84] (Table 6).

### 3.4. Combination of Enzymes with Other Technologies

Chemat et al. put forward the concept of “green extraction of natural products” in 2012: “Green Extraction is based on the discovery and design of extraction processes that will reduce energy consumption, allow the use of alternative solvents and renewable natural products, and ensure safety and high quality extracts/products”. The green extraction technologies can complete the extraction in a short time with high reproducibility, reduced solvent consumption, simplified operations, high purity, elimination of wastewater post-treatment, and low energy consumption [91]. To further improve the efficiency and quality of traditional medicine extraction, some studies have combined enzyme-assisted extraction with green extraction technologies such as membrane separation, ultrasonic extraction, microwave, and macroporous resin separation. 

Radio frequency (RF) is a rapid heating method (3 kHz to 300 MHz) that can deeply penetrate materials without leaving chemical residues. Jiang et al. proposed a novel RF heating-assisted enzymatic extraction method and determined that the optimal extraction conditions were equimolar amounts of cellulase and pectinase at an enzyme concentration of 1.0%, 50% ethanol, a liquid–solid ratio of 50 mL/g, and pH 4, with a radiofrequency pretreatment at 40 °C for 10 min with an electrode gap of 5 cm. The results show that the crude product (26.55%) and anthocyanin yields (50.87 mg cyanidin-3-O-glucoside equivalents/100 g) were higher compared to hot water, acidified ethanol, and enzyme (pectinase and cellulase) extraction [92]. 

The principle of the microwave extraction method is to use the huge penetration of electromagnetic waves (300 MHz to 300 GHz) to increase the internal pressure of the cell above its ability to withstand so that the effective ingredients flow out of the cell. In this extraction process, both the extraction solvent and material have varying degrees of influence on the dielectric constant and loss factor that affect the extraction efficiency. Microwave extraction can quickly heat up, shorten extraction time, and improve efficiency, and it meets environmental protection requirements. Combining enzymatic and microwave extraction can improve extraction efficiency and rate under mild conditions. Yang et al., evaluated the efficiency of microwave-assisted enzyme extraction in extracting corilagin (CG) and geraniin (GE). They found that after 9 min of treatment with cellulase (3600 U/g) and irradiation (500 Mpa) at pH 5.2 and 33 °C, extraction yields of CG and GE were increased by 64.01% and 72.95%, achieving 6.79 and 19.82 mg/g, respectively [93]. 

Ultrasonic extraction uses the mechanical, cavitation, and thermal effects of ultrasonic vibration to improve the diffusion of solvents and accelerate the dissolution of effective ingredients. Enzymatic hydrolysis–ultrasonic coupling is an emerging technology with a high extraction rate and efficiency, saving both time and energy during the auxiliary extraction of natural active substances, and can effectively increase the extraction rate of various bioactive substances. Huang et al. optimized a cellulase–ultrasonic-assisted method to extract flavonoids from laver residue. They found that the optimal extraction conditions were 51.14% ethanol and a liquid–solid ratio of 20.52 mL/g at a constant 45 °C. An ultrasonic treatment time of 60 min with enzymatic hydrolysis at pH 5.303 for 2 h using 70 mg/g of enzyme and a crushed mesh size of 0.355–0.85 mm gave a maximum yield of 14.76% [94]. Hua et al. studied the extraction of *Panax notoginseng* saponin (PNS) and its antioxidant activity. PNS was extracted by enzymatic hydrolysis and ultrasonic treatment. The results showed that the best ultrasonic treatment parameter was a 1:15 ratio of material to liquid, 70% ethanol, and 35 min ultrasonic time. The optimal conditions for enzymatic hydrolysis were 60 min at 40 °C and pH 4.0 with 2.2% cellulase. Under these conditions, PNS extraction reached 1.795% [95]. Guo et al. studied the cellulase–ultrasonic wave method for extracting polysaccharides from *Lenzites betulina* and optimized extraction conditions by RSM. They found that after 180 min of reaction in 0.8% cellulase with a pH of 4.5 at 60 °C and ultrasonic treatment (300 W) at 45 °C for 20 min, the maximum extraction yield of *L. betulina* polysaccharides was 13.64 ± 0.09% [96]. 

HHP-assisted enzyme extraction uses pressure to enhance enzyme activity and improve wall-breaking efficiency. It can also be used to intensify the mass transfer process, increase the mass transfer rate, promote the dissolution of polysaccharides, and shorten the extraction time, thereby increasing polysaccharide yields. Compared to other extraction technologies, its advantages are high extraction efficiency, mild conditions, low cost, and easy continuous operation. Sunwoo et al. evaluated the effect of HHP combined with enzymatic hydrolysis to extract ginsenoside from fresh ginseng root (*Panax ginseng* CA Myer). Ginseng roots were decomposed by cellulase or β-amylase with HHP (100 MPa) at 50 °C for 12 h, which increased the production of total saponins, panaxadiols, and metabolites. The total saponin production in HHP-EH with cellulase was 40.2 mg/mL, which was significantly higher than that with β-amylase (36.1 mg/mL) (*p* < 0.05) [97]. Palaniyandi et al. used a combination of polysaccharide hydrolase and HHP to extract ginsenosides rich in ginsenosides Rg1 and Rb1. Their study showed that the combined treatment of cellulase, amylase, and pectinase for 12 h at a pressure of 100 MPa, pH 4.8, and 45 °C can increase the levels of Rg1 and Rb1 in the extract [98]. 

It is also possible to combine enzyme-assisted extraction with multiple physical technologies to further optimize the extraction effect. Li et al. proposed a continuous process combining ultrasonic, microwave, and enzymatic hydrolysis to assist the extraction of genipin from *Eucommia ulmoides* bark. A mixture of dry bark powder and deionized water was irradiated under a 500 W microwave for 10 min and then was incubated in a 0.5 mg/mL cellulase solution (pH 4.0) for 24 h at 40 °C. Ultrasound was performed for 30 min after the addition of ethanol, and the final genipin yield could reach 1.71 μmol/g [99].

At present, ultrasonic- and microwave-assisted extraction and other ultra-new technologies are widely used to extract bioactive ingredients. Compared with the traditional extraction process, they have unique application characteristics and advantages. On this basis, combined with the damaging effect of cellulase on the cell wall barrier, the biologically active ingredients can be dissolved more easily. At present, there are relatively few studies on extraction methods that combine cellulase-assisted extraction and various physical technologies. For this type of method, the complex extraction conditions need to be determined through experiments, and expensive equipment is required. Therefore, it is limited to laboratory research and lacks in-depth research and transformational applications (Table 7).

## 4. Conclusions and Outlook

Cellulase-assisted extraction technology provides new ideas and methods for producing and researching bioactive medicinal ingredients and can significantly increase the extraction rate of effective ingredients and overcome the complex procedures and time consumption of traditional methods. Cellulase was initially usually used alone or in combination with other enzymes for the pretreatment of plant materials. Using some appropriate enzymes (including cellulase, hemicellulase, and pectinase) on plant cells can degrade cellulose, hemicellulose, pectin, and other substances in the cell wall and the intercellular space. It can destroy the dense structure of the cell wall to reduce the mass transfer resistance of the cell wall and other mass transfer barriers. In general, treating medicinal materials with complex enzymes is better than cellulase alone. With the advancement of technology and research, using physical methods, such as ultrasound, microwave, and HHP, in combination with enzymatic hydrolyses to extract bioactive substances has become a viable approach. The reaction conditions of enzymatic hydrolysis are mild, which can maintain the conformation of the natural product without destroying its three-dimensional structure and biological activity and will also reduce pollutant emissions. Although enzymatic hydrolysis also has limitations and requires high industrial application conditions, the broad application prospects and economic benefits should encourage researchers and engineers to develop further and optimize related manufacturing techniques. 

With the continuing research on cellulase-assisted extraction technology, the limitations of cellulase have gradually emerged. Its high cost restricts the development of this technology, and finding new cellulases is a way to reduce production costs. Molecular biology methods and DNA recombination technology can be used to screen special microbial strains for enzyme-producing genes (including new enzymes in extreme environmental conditions) and chemical methods or genetic engineering can be used to modify existing enzymes to construct specifically engineered bacteria. As enzymes are extremely sensitive to reaction conditions, it is necessary to determine the optimum temperature, pH, and reaction time to maximize cellulase activity throughout experiments. The influence of enzyme concentration, substrate concentration, agonists, and inhibitors should also be considered. At the moment, most of the cellulases used are processed by heating and inactivation and cannot be recovered. This not only increases cost but also has an impact on extract safety and effectiveness due to cellulase residue. There are some profound problems that need to be solved: whether the enzyme residue will degrade, precipitate, or form a complex with the bioactive ingredients in the preparation; whether it will affect the quality and quantity of the bioactive ingredients; whether it will produce adverse reactions; and whether it will affect the quality of the preparation or interfere with detection and affect safety or effectiveness. Research into nonaqueous mediator enzyme reactions and immobilized enzymes is an effective way to improve enzyme stability and potential recycling. In conclusion, cellulase-assisted extraction technology cannot solve all the problems in the extraction of bioactive ingredients. As a new technology, it must be applied in conjunction with other technologies to allow exploitation of its advantages, and we consider that these issues will become the focus of future research.

## Figures and Tables

**Table 1:** Methods for mining new cellulase-producing strains or cellulase gene fragments.

Gene Source	Sample Type	Types of Enzymes	Method of Mining Genes	Year	Reference
*Thalassobacillus* sp. LY18	saline soil of Yuncheng Salt Lake, China	alkaline endoglucanase	pure culture	2012	[11]
*Bacillus licheniformis* AMF-07	Kerman hot spring	cellulase	pure culture	2016	[12]
*Paenibacillus* sp. CKS1	soil	cellulase	pure culture	2016	[13]
*Trichoderma harzianum* LZ117	surface of bryophyte on a stone in Tibet	cellulase	pure culture	2019	[14]
unknown source	soil	endoglucanase (Cel5A)	functional metagenomics	2006	[15]
unknown source	biogas plant	cellulase (CelA2, CelA3)	functional metagenomics	2012	[16]
	elephant feces	cellulase (CelA84)			
unknown source	*Ascophyllum nodosum* from the foreshore in Roscoff	cellulase (CellMM5.1)	functional metagenomics	2014	[17]
unknown source	soil	endoglucanase (Cel5Rα)	functional metagenomics	2016	[18]
unknown source	outflow of a hot spring in Grensdalur, Iceland	cellulase (CelDZ1)	shotgun metagenomics	2016	[19]
unknown source	anaerobic beer lees	cellulase (cel7482, cel3623, cel36)	shotgun metagenomics	2016	[20]
unknown source	Black Slug *Arion ater* from North Cheshire	β-glucosidase	shotgun metagenomics	2017	[21]
unknown source	hepatopancreas of a female *Cherax quadricarinatus*	endoglucanase	PCR	1999	[22]

**Table 2:** Common methods for predicting flexible regions in proteins.

Method	Instructions	Way to Obtain
molecular dynamics simulation	Examine the flexibility of protein at the atomic level.	Gromacs software
B-FITTER	Calculate the B-factor value of all atoms in an amino acid, and then take the average to obtain the B-factor value of this residue.	http://www.kofo.mpg.de/en/research/organic-synthesis (accessed on 2 July 2021)
FoldUnfold	Calculate the number of interaction forces involved in each amino acid residue to determine whether a region is in a folded or unfolded state.	http://bioinfo.protres.ru/ogu/ (accessed on 2 July 2021)
PredyFlexy	Combine B-factor with the movement state of amino acid residues during molecular dynamics simulation to analyze.	http://www.dsimb.inserm.fr/dsimb_tools/predyflexy/ (accessed on 2 July 2021)
FlexPred	Use algorithm SVM to predict the flexibility of residues.	http://flexpred.rit.albany.edu (accessed on 2 July 2021)
HINGEprot	Predict the hinge region of a protein.	http://bioinfo3d.cs.tau.ac.il/HingeProt/ (accessed on 2 July 2021)

**Table 3:** Methods for stiffening flexible regions.

Type of Enzyme	Gene Source	Influencing Factor	Software	Methods to Improve Thermal Stability	Year	Reference
cellulase (*Ta*Cel45)	*Thielavia arenaria* XZ7	disulfide bond		Introduction of disulfide bonds into flexible regions can stiffen it and improve protein thermostability.	2018	[42]
endoglucanase (PvCel5A)	*Penicillium verruculosum*	proline	RosettaDesign, HotSpot Wizard, PopMuSiC, UniProt	Introduction of proline can reduce the conformational entropy of main chain and improve protein thermal stability.	2019	[43]
1,4-α-glucan branching enzyme		*C*-terminal flexible area		Shortening the flexible area can increase its rigidity and protein thermal stability.	2018	[44]
mannanase (Man1312)	*Bacillus subtilis* B23	*N*-terminal flexible area	SWISS-MODE, Protein Structure Validation Software, PyMOL23 Swiss-PdbViewer, POODLE		2016	[45]
alkaline, mesophilic endo-1,4-β-glucanase	*Bacillus sp.* strain KSM-64	salt bridge	InsightII/Discover software package	Introduction of salt bridges can increase protein thermal stability.	2001	[46]
cellobiohydrolase (Cel7A)	*Trichoderma reesei* (anamorph *Hypocrea jecorina*)	*N*-glycosylation		Glycosylated proteins are less likely to aggregate and prevent hinges or links from being affected and their thermal stability can be improved.	2017	[47]
cellulase (GtCel5)	*Gloeophyllum trabeum* CBS 900.73	loop structure	BLAST, GENSCAN Web Server, SignalP 3.0, NetNGlyc 1.0 Server Vector NTI Suite 10.0, MEGA 4.0	Directed mutations in a flexible loop can improve protein thermal stability.	2018	[48]

**Table 4:** Structural modification of bioactive components by cellulase during extraction.

Types of Enzymes	Physical Technology	Substrate	Product	Year	Reference
cellulase-12T		WGE	convert ginsenoside Rb1 to Rg3	2009	[63]
cellulase		fruit of *Gardenia jasminoides* Ellis	convert geniposide to genipin	2013	[64]
penicillium, decumbens, cellulase		*Ginkgo biloba* leaves	transglycosylate flavonol aglycones into glucosides	2011	[65]
cellulase, cellobiase	HHP	*Panax ginseng*	transform major ginsenosides into ginsenoside Rd	2015	[66]

**Table 5:** Application of cellulase in the extraction of bioactive components.

Type of Enzyme	Substrate	Product	Year	Reference
cellulase	flos lonicerae	chlorogenic acid	2002	[72]
cellulase	*Eucommia ulmoides* Oliv.	phenolic compounds	2009	[73]
cellulase	*Taxus chinensis*	paclitaxel and related compounds	2009	[74]
cellulase	*Hypericum perforatum* L.	naphthodianthrones and pseudohypericin	2012	[75]
cellulase	*Eucommia ulmoides* leaves	aucubin	2012	[76]
cellulase	*Dendrobium chrysotoxum*	polysaccharides	2015	[67]
cellulase	*Eucommia ulmoides*	chlorogenic acid	2016	[68]
cellulase	Radix *Angelica sinensis*	Z-ligustilide	2017	[69]
cellulase	*Astragalus*	APS	2019	[70]
cellulase	*Hovenia dulcis*	reducing sugars	2019	[71]

**Table 6:** Application of mixed enzymes (containing cellulase) in the extraction of bioactive ingredients.

Types of Enzymes	Substrate	Product	Year	Reference
protease, cellulase	*Salvia miltiorrhiza*	rosmarinic acid	2020	[77]
cellulase, pectinase, papain	*Agaricus blazei Murrill*	polysaccharides	2013	[85]
cellulase, pectinase	licorice	glycyrrhizinate	2013	[86]
cellulase, pectase, papain	*Astragalus membranaceus*	APS	2015	[78]
cellulase, α-amylase	brown seaweeds	phlorotannins	2016	[79]
cellulase, papain, pectinase	*Lentinus edodes*	polysaccharides	2016	[80]
cellulase, pectinase, xylanase	white hyacinth bean	polysaccharide	2016	[81]
cellulase, lysozyme	microalgae	protein	2017	[87]
viscozyme, termamyl, cellulase	*Panax notoginseng*	ginsenoside Rb1 and Rg3	2018	[88]
cellulase, α-amylase	*Panax ginseng* Meyer	polysaccharides	2018	[82]
proteases, cellulase	pumpkin seeds	pumpkin seed oil	2019	[89]
cellulase, xylanase	*Echinacea angustifolia* L.	polysaccharides and antioxidants	2019	[90]
cellulase, hemicellulase	*Mentha arvensis* L.	essential oil	2020	[83]
cellulase, alginate lyase	brown seaweeds	fucoidans	2020	[84]

**Table 7:** Combined application of cellulase or mixed enzymes and physical techniques in the extraction of bioactive ingredients.

Types of Enzymes	Physical Technology	Substrate	Product	Year	Reference
cellulase	microwave	*Geranium sibiricum* Linne	CG, GE	2010	[93]
cellulase	ultrasonic; microwave	*Eucommia ulmoides* bark	Genipin	2015	[99]
cellulase	ultrasonic	*Illicium verum*	flavonoids	2016	[94]
cellulase	ultrasonic	Panax notoginseng	PNS	2016	[95]
cellulase	ultrasonic	*Lenzites betulina*	polysaccharides	2019	[96]
cellulase, β-amylase	HHP	*Panax ginseng* CA Myer	ginsenosides	2014	[97]
cellulose, β-glucosidase	pulsed electric field	ginseng	ginsenosides, polyphenols, flavonoids	2018	[100]
cellulase, amylase, pectinase	HHP	ginseng	ginsenosides Rg1 and Rb1	2017	[98]
cellulase, pectinase	RF	*Akebia trifoliata* (Thunb.) Koidz flowers	anthocyanins	2020	[92]
